# A Case of Confusion in an Obese Patient Treated With Daptomycin: Neurotoxicity

**DOI:** 10.7759/cureus.62254

**Published:** 2024-06-12

**Authors:** Rova Malala Fandresena Randrianarisoa, Olivia Raulin, Anthony Merlin, Mathilde Tonnelier, Anne-Lise Lecapitaine

**Affiliations:** 1 Internal Medicine, University Hospital Joseph Raseta Befelatanana, Antananarivo, MDG; 2 Medical Biology, Compiègne-Noyon Hospital, Compiègne, FRA; 3 Internal Use Pharmacy, Compiègne-Noyon Hospital, Compiègne, FRA; 4 Infectious and Tropical Diseases, Compiègne-Noyon Hospital, Compiègne, FRA

**Keywords:** obesity, neurotoxicity, daptomycin, confusion, adverse effects

## Abstract

Daptomycin (DAP) is a cyclic lipopeptide antibiotic with bactericidal activity against gram-positive bacteria. The most common adverse reaction is myotoxicity characterized by rhabdomyolysis. Other reported adverse reactions include gastrointestinal symptoms, skin lesions, bleeding, and pulmonary involvement. Neurotoxicity is rare and its mechanism remains partially elucidated. We report a case of confusion consistent with DAP-induced neurotoxicity. A 73-year-old obese man was treated with DAP 9 mg/kg for methicillin-resistant *Staphylococcus aureus *(MRSA) bacteremia associated with foot osteitis and cervical posterior inter-apophyseal arthritis. On the fifth day of treatment, he developed spatial disorientation, and serum DAP concentrations were very high. DAP-induced neurotoxicity was suggested. His neurological status returned to normal after treatment was stopped. This observation describes a relationship between confusion and DAP that is favored by obesity. Clinicians should be alert for neurologic disorders associated with DAP. It is prudent to reduce doses in obese patients.

## Introduction

Daptomycin (DAP) is a cyclic lipopeptide antibiotic derived from the fermentation of *Streptomyces roseosporus*. It has bactericidal activity against gram-positive bacteria, particularly methicillin-resistant *Staphylococcus aureus* (MRSA) and vancomycin-resistant enterococci [1,2]. According to the European Medicines Agency, DAP is indicated for the treatment of complicated skin and soft tissue infections, infective endocarditis of the right heart, and susceptible bacteremia.

The most common adverse reaction of DAP is muscle toxicity characterized by elevated creatine phosphokinase (CPK). Other adverse reactions have been reported, including gastrointestinal symptoms, skin lesions, bleeding, and pulmonary involvement [3-5]. The occurrence of neurological disorders is rare and their mechanism remains partially elucidated. Cases of encephalopathy and neuropsychiatric disorders have been reported in the literature [6-8]. To shed new light on the side effects of DAP, we report a case of confusion during DAP treatment, suggesting neurotoxicity.

## Case presentation

A 73-year-old man with hypertension and diabetes mellitus was admitted for right-sided heart failure. His medical history was significant for obesity (weight: 150 kg, height: 176 cm, and BMI: 48.42 kg/m^2^), atrial fibrillation, stage 3b chronic kidney disease, and a left transfemoral amputation. He has had a pacemaker for three years. One year ago, he was admitted to the hospital with MRSA osteitis of the right foot. He had no psychiatric history and was a moderate drinker. His usual medications were semaglutide, apixaban, atorvastatin, furosemide, tramadol, pregabalin, tamsulosin, and lansoprazole.

On admission, hemodynamic variables were normal. He reported no fever and was in good general condition. His Glasgow score was 15/15, and his neurological examination was normal. Cardiac and pulmonary auscultation were normal. Examination revealed painless edema of the right leg, venous leg ulcers, and a wound on the fifth radius of the right foot. The results of the laboratory tests are summarized in Table 1.

**Table 1:** Biological blood test results CPK, creatine phosphokinase; DAP, daptomycin

	Normal value	On admission	On day 5 of DAP
Hemoglobin (g/dL)	12.9-16.7	8.3	9.5
Mean corpuscular volume (fL)	83.0-97.0	74.5	74.6
White blood cell count (G/L)	4.00-11.00	7.37	7.11
Platelet (G/L)	140-385	291	258
Reticulocyte (G/L)	30-120	63	79.1
Ferritin (µg/L)	30-300	42	-
Soluble transferrin receptor (mg/L)	0.83-1.76	3.09	-
Transferrin saturation coefficient (%)	23-43	3	-
Glucose level (mmol/L)	<11.1	7.8	6.8
Glycated hemoglobin (%)	4.4-6.5	6.4	-
Sodium (mmol/L)	135-144	139	146
Potassium (mmol/L)	3.5-5.1	3.7	3.9
Creatinine (µmol/L)	60-110	151	138
Urea (mmol/L)	<11.9	12.5	8.4
Calcium (mmol/L)	2.12-2.60	-	2.37
Albumine (g/L)	35-48	32	-
Total bilirubin (µmol/L)	<21	24	37
Aspartate aminotransferase (UI/L)	5-45	14	18
Alanine aminotransferase (UI/L)	<41	9	10
Alkaline phosphatases (UI/L)	40-130	128	129
Gamma-glutamyl transferase (UI/L)	<60	86	74
CPK (UI/L)	20-200	-	53
NT-proBNP (ng/L)	<300	11894	-
C-reactive protein (mg/L)	<10	186	142
Thyroid-stimulating hormone (mU/L)	0.27-5.50	-	2.59

A complete blood count revealed moderate anemia. Blood ionogram and liver function tests were normal. Serum creatinine was 151 µmol/L with a clearance of 38 mL/min (versus a baseline creatinine of 150 µmol/L). Serologies for HIV, hepatitis B, and hepatitis C were negative. Blood culture was positive for MRSA (Table 2). Urine bacteriology was negative.

**Table 2:** Blood culture and antibiogram results MIC, minimal inhibitory concentration; R, resistant; S, sensitive

Antibiotic	Sensitivity	MIC E-test (mg/L)
Penicillin G	R	-
Oxacillin	R	-
Cefazolin	R	-
Ceftaroline	S	0.750
Gentamycin	S	-
Tobramycin	S	-
Kanamycin	S	-
Tetracycline	S	-
Erythromycin	R	-
Clindamycin	R	-
Pristinamycin	S	-
Quinupristin/dalfopristin	S	-
Cotrimoxazole	S	-
Norfloxacın	R	-
Rifampicin	S	-
Fusidic acid	S	-
Fosfomycin	S	-
Linezolid	S	-
Vancomycin	S	0.500
Teicoplanin	S	0.250
Daptomycin	S	0.19
Dalbavancin	S	0.032

**Figure 1:**
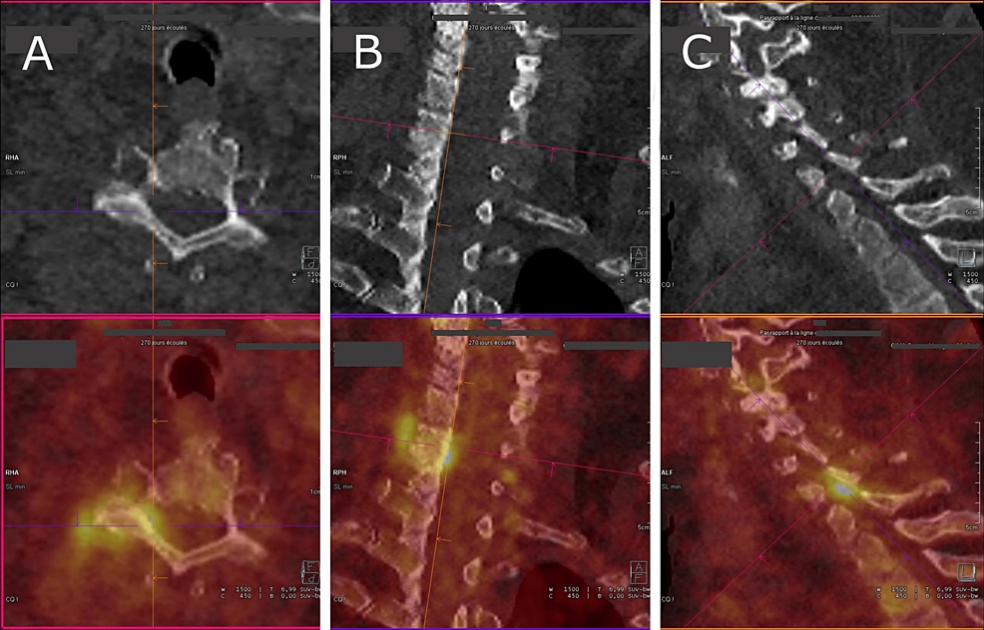
PET scan showing hypermetabolic activity in the right inter-apophyseal joint C6-C7 (SUV=5) A: axial view; B: coronal view; C: sagittal view PET, positron emission tomography

**Figure 2:**
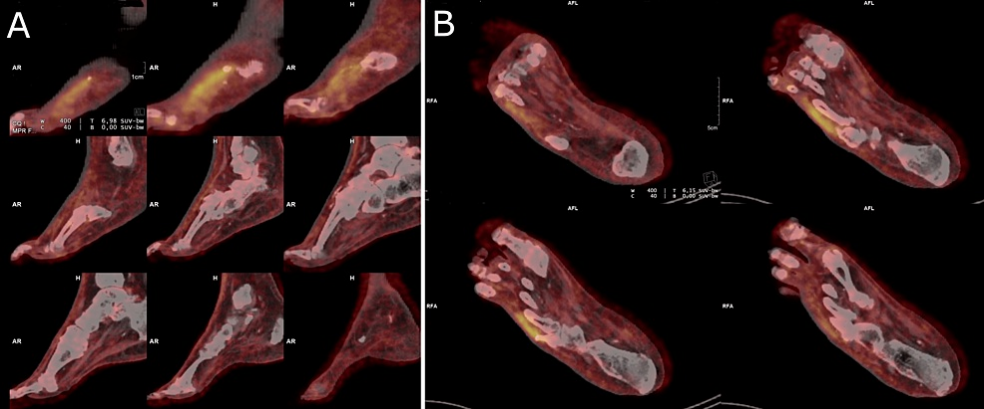
PET scan showing hypermetabolic activity of the fifth radius of the right foot A: sagittal view; B: axial view PET, positron emission tomography

Transthoracic echocardiography showed a dilated right ventricle and preserved systolic ejection function. Transesophageal echocardiography showed no valvular vegetation or intracavitary thrombus, and the pacemaker was intact. Computed tomography (CT) pulmonary angiography was normal. Positron emission tomography (PET) scan showed hypermetabolic activity in the right inter-apophyseal joint C6-C7 (Figure 1) and hypermetabolism of the bone stump and soft tissues of the fifth radius of the right foot (Figure 2). There was no hypermetabolic activity of the heart or pacemaker.

The diagnosis was MRSA bacteremia associated with osteitis of the fifth radius of the right foot and inter-apophyseal arthritis C6-C7. The anemia was multifactorial: iron deficiency, inflammatory, and renal. Renal insufficiency was stable. He was treated with furosemide and a packed red blood cell transfusion was performed. As soon as blood cultures were positive, treatment with DAP 9 mg/kg (1300 mg) once daily was started. Subsequent blood cultures were negative from day three of antibiotic treatment.

**Figure 3:**
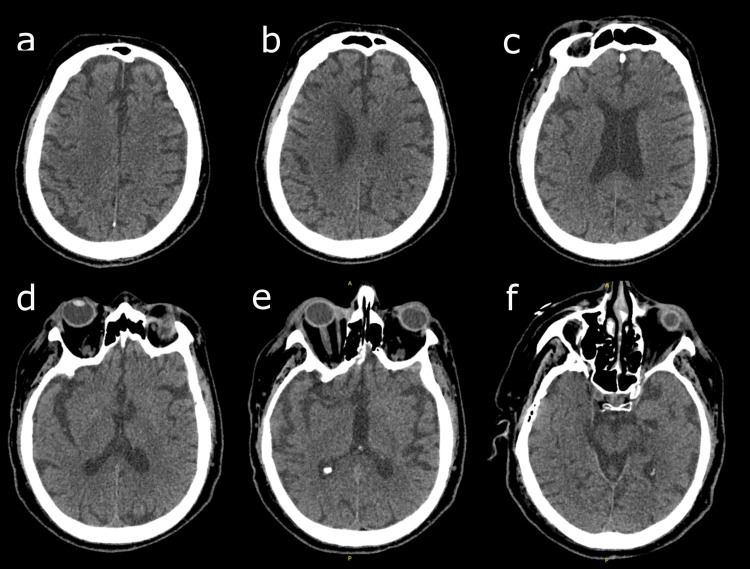
Axial brain CT scan showing no specific abnormality Axial CT scan slices, from top (a) to bottom (f) CT, computed tomography

On day five of treatment, spatial disorientation occurred. Hemodynamic variables were stable and blood glucose levels were normal. There was no urinary retention or constipation. Examination revealed no focal neurological deficit or meningeal syndrome. Osteotendinous reflexes, cranial nerves, and muscle strength were preserved. The signs of congestive heart failure resolved. Hemoglobin level had increased to 9.5 g/dL. Blood ionogram and renal and liver function tests were stable. CPK was not elevated and TSH was normal (Table 1). Arterial blood gases had pH: 7.45; PaCO_2_: 45 mmHg; PaO_2_: 66 mmHg; HCO_3_: 30.8 mmol/L; total CO_2_: 32.2 mmol/L; and saturation: 93%. Tramadol and pregabalin were discontinued in the iatrogenic hypothesis. CT scan of the brain showed no significant abnormalities other than age-related cortico-subcortical atrophy (Figure 3).

**Figure 4:**
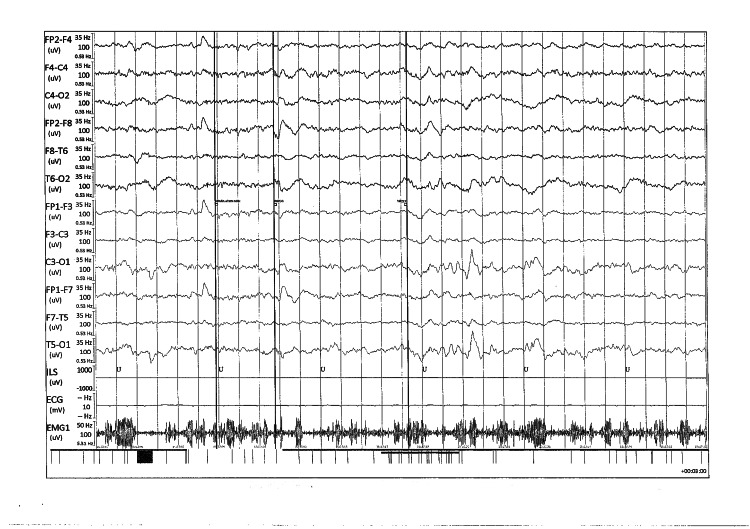
EEG traces showing slowed, poorly organized, symmetrical, and reactive background activity with theta rhythms and bursts of slow delta waves of diffuse projection EEG, electroencephalogram

Electroencephalogram (EEG) results showed a marked slowing of poorly organized background activity without focal or epileptiform abnormalities, subject to numerous artifacts (Figure 4).

The persistence of confusion led to the hypothesis of a DAP-induced neurological disorder. DAP was replaced by vancomycin on day eight of treatment. The residual plasma DAP concentration was 49.6 mg/L 48 hours after stopping treatment. The patient’s neurological status returned to normal within 24 hours of discontinuing DAP. To facilitate patient autonomy, vancomycin was replaced with a combination of dalbavancin and doxycycline for a total of six weeks of treatment. This adverse event was reported to the national pharmacovigilance system.

## Discussion

The approved first-line antibiotics for MRSA infections are vancomycin and DAP. DAP offers advantages in terms of ease of use (once-daily administration, reduced genotoxicity and nephrotoxicity, and no need for pharmacologic follow-up) [1,9]. In recent recommendations, guidelines support the use of high-dose DAP (≥8 mg/kg) for severe MRSA infections such as bacteremia, infective endocarditis, and osteoarticular infections [10,11].

DAP exerts its bactericidal activity by binding to the bacterial cell membrane, causing depolarization of the membrane potential. This leads to intracellular inhibition of nucleic acid and protein synthesis. A special feature of DAP is its high efficacy against gram-positive bacteria in biofilms [2,9].

Myotoxicity is the most common side effect and is characterized by elevated CPK levels. This toxicity presents as myalgias and rhabdomyolysis and often leads to discontinuation of treatment [3]. Regular CPK monitoring is recommended in treated patients. Other observed adverse effects include hyperthermia, gastrointestinal disturbances (nausea, vomiting, and diarrhea), and skin manifestations (pruritus, urticaria, burns) [4]. Acute eosinophilic pneumonia is a potential adverse event that occurs two to four weeks after initiation of treatment. It is explained by the interaction of DAP with pulmonary surfactant [5].

DAP-induced neurotoxicity is uncommon. Central and peripheral nervous system involvement and psychiatric disorders have been described [12]. Bitar de Zayas-Enriquez et al. reported a case of reversible posterior encephalopathy manifested by tonic-clonic seizures within 50 minutes of the first dose of DAP [7]. Scolari et al. reported a case of upper limb myoclonus on the sixth day of treatment [13]. Afriyie et al. reported a case of acute delirium with confusion and hallucinations [8]. In a study by Teng et al., only nine cases of delirium were observed among 5460 patients treated with DAP [14]. In our case, the patient developed spatial disorientation on the fifth day of treatment. We ruled out possible metabolic causes and discontinued drugs that could cause confusion. Given the lack of response, DAP-induced neurotoxicity was suspected. The disappearance of confusion after drug discontinuation, the high plasma concentration of DAP even 48 hours after discontinuation, and the normal CPK level supported our hypothesis. Furthermore, using the probability of adverse drug reaction algorithm developed by Naranjo et al., the score was eight, indicating probable causality [15].

The mechanism of DAP-induced neurotoxicity is poorly understood. It is thought to be caused by interaction with gamma-aminobutyric acid and dopamine D2 receptors and alteration of the membrane wall of endothelial and neuronal cells [7,14,16]. Renal insufficiency and obesity are risk factors for overdose and toxicity [17]. DAP is primarily excreted by the kidneys. Patients with a clearance of less than 30 mL/min require dose adjustment [18]. In obese patients, it is prudent to use the adjusted body weight [1,19]. In our patient, DAP dosage was initially based on actual body weight, resulting in overdosage and likely toxicity. Some authors have suggested that residual concentrations above 20 mg/L are associated with a greater risk of toxicity, which may argue for pharmacologic monitoring of DAP in patients at risk of overdose (obese and with renal insufficiency) [20].

## Conclusions

This observation describes a close association between confusion and DAP treatment. Given the lack of an alternative diagnosis and the rapid resolution of confusion after discontinuation of the drug, DAP-induced neurotoxicity is the most likely diagnosis. Clinicians should be alert to neurologic disorders associated with DAP. In obese patients, it is prudent to reduce doses and not use actual body weight. Further studies are needed to understand the pathophysiologic mechanisms of this neurotoxicity.
